# Canine leishmaniosis and its relationship to human visceral leishmaniasis in Eastern Uzbekistan

**DOI:** 10.1186/1756-3305-4-58

**Published:** 2011-04-13

**Authors:** Dmitriy A Kovalenko, Shavkat A Razakov, Evgeny N Ponirovsky, Alon Warburg, Rokhat M Nasyrova, Valentina I Ponomareva, Aziza A Fatullaeva, Abdelmajeed Nasereddin, Eyal Klement, Mohammad Z Alam, Lionel F Schnur, Charles L Jaffe, Gabriele Schönian, Gad Baneth

**Affiliations:** 1 Isaev Institute, Samarkand, Uzbekistan; 2 Sechenov Medical Academy, Moscow, Russia; 3 IMRIC, Hebrew University - Hadassah Medical, Israel; 4 School of Veterinary Medicine, Hebrew University, Israel; 5 Charité University Medicine, Berlin, Germany

## Abstract

**Background:**

The Namangan Region in the Pap District, located in Eastern Uzbekistan is the main focus of visceral leishmaniasis (VL) in Uzbekistan. In total, 28 cases of human VL were registered during 2006-2008 in this region. A study on the epidemiology of VL in this area was carried out in 2007-2008 in the villages of Chodak, Oltinkan, Gulistan and Chorkesar located at elevations of 900-1200 above sea level.

**Results:**

A total of 162 dogs were tested for *Leishmania *infection. Blood was drawn for serology and PCR. When clinical signs of the disease were present, aspirates from lymph nodes and the spleen were taken. Forty-two dogs (25.9%) had clinical signs suggestive of VL and 51 (31.5%) were sero-positive. ITS-1 PCR was performed for 135 dogs using blood and tissue samples and 40 (29.6%) of them were PCR-positive. Leishmanial parasites were cultured from lymph node or spleen aspirates from 10 dogs.

Eight *Leishmania *strains isolated from dogs were typed by multi-locus microsatellite typing (MLMT) and by multilocus enzyme electrophoretic analysis (MLEE), using a 15 enzyme system. These analyses revealed that the strains belong to the most common zymodeme of *L. infantum*, i.e., MON-1, and form a unique group when compared to MON-1 strains from other geographical regions.

**Conclusions:**

The data obtained through this study confirm the existence of an active focus of VL in the Namangan region of Uzbekistan. The fact that *L. infantum *was the causative agent of canine infection with typical clinical signs, and also of human infection affecting only infants, suggests that a zoonotic form of VL similar in epidemiology to Mediterranean VL is present in Uzbekistan.

## Background

**Figure 1:**
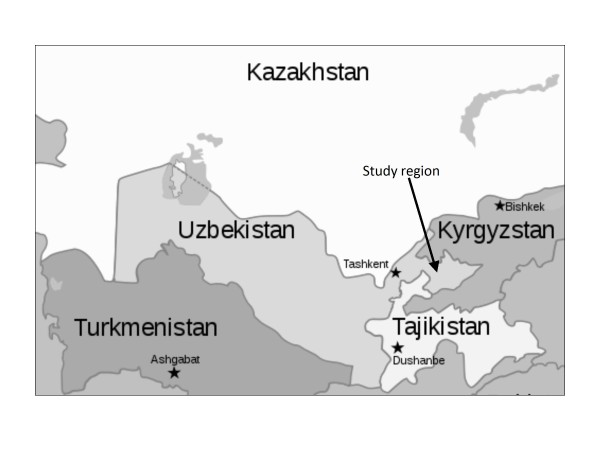
**Map of Central Asia.** A map of Central Asia showing the study area located in Eastern Uzbekistan.

Visceral leishmaniasis (VL) has been reported for more than half a decade from the Fergana valley and the foothills surrounding this valley in the eastern part of the central Asian republic of Uzbekistan [1]. The Pap district in the Namangan Region, located in the Western Fergana is a major stable focus of VL which borders Tajikistan and Kyrgyzstan (Figure 1) where VL has also been reported [2,3]. In total, 28 cases of human VL were registered during 2006-2008 in the Namangan region of which all were children, all but one younger than four years old [4]. The causative agent of the disease in this area is *Leishmania infantum *[5], and the suspected vector is the sand fly *Phlebotomus longiductus *[3].

Studies on the epidemiology of VL in this area were carried out in 2007-2008 in the villages of Chodak, Oltinkan, Gulistan and Chorkesar. A study on VL in the human population in this region found that 11% of 432 children were seropositive to *L. infantum *antigen by the enzyme-linked immunosorbent assay (ELISA) and *L. infantum *DNA was amplified from Giemsa-stained slides of bone marrow from eight VL patients [4,5].

Several sand fly species were found in a previous entomological survey in the same area with *Phlebotomus sergenti *being the most prevalent species (46%), followed by *P. papatasi *(18.8%), *P. longiductus *(15.5%), *P. alexandri *(10.3%) and other species [3]. The suspected local vector of VL in the Namangan region is *P. longiductus *as it was found to transmit VL caused by *L. infantum *elsewhere in Central Asia [3,6]. An entomological study carried out in the Namangan region concomitantly with the human disease study in 2007 and 2008 included the analysis of 10,000 sand flies and was in general agreement with species composition found by Maroli et al. in 2001 [[3], Ponirovskii and Warburg, unpublished data].

The aim of this study was to investigate the presence of canine leishmaniosis in the Namangan human VL focus and to evaluate the possible role of domestic dogs as the disease reservoir.

## Methods

### Study area

**Figure 2:**
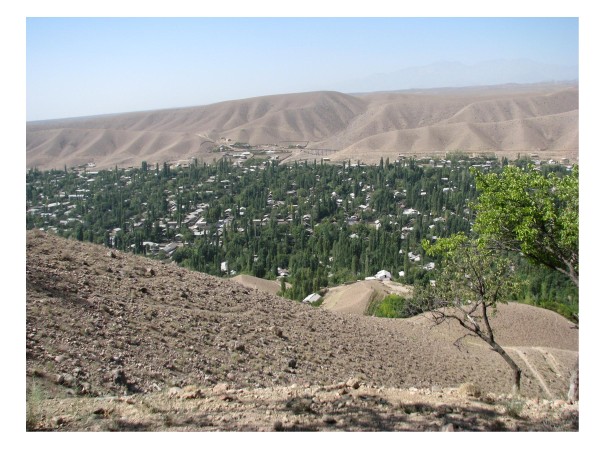
**General view of Chodak**. An overview of the Chodak village, a focus of visceral leishmaniasis in the Namangan region of Uzbekistan, situated along the Chodaksoj river and surrounded by an arid mountainous area.

The study was carried out in the villages of Chodak, Oltinkan, Gulistan and Chorkesar situated along the Chodaksoj river and the nearby village of Chorkesar. These villages are located at elevations of 900-1200 above sea level in the Pap District of the Namangan region. The study area is mountainous and semi-arid and the villages of Chodak, Oltinkan, and Gulistan are located in a wide canyon with small garden plots and trees irrigated by water pumped from the river (Figure 2). The population is composed mainly of farmers who grow field crops, fruit and vegetables and raise cattle. Cattle graze in the highlands during the warmer months accompanied by local herders and dogs. Domestic habitations are typically enclosed by a stone or mud wall, and contain gardens ranging from 50 to 1000 square meters, animal quarters, and separate living quarters for humans. The total population in the four villages was 33,726 in 2008 of which 35.6% were children under 10 years old (Table 1). Almost all families have at least one dog used mainly for guarding, and herding. Dogs remain outside homes and are typically not allowed to enter human living quarters.

**Table 1:** The total population and number of children in the villages included in the study in 2007-2008 (Source - Uzbekistan Ministry of Health).

Village	Population	Children up to 10 years
Chodak	16,450	4,859 (29.53%)
Oltinkan	3,775	1,482 (39.25%)
Gulistan	1,849	550 (29.74%)
Chorkesar	11,652	5,103 (43.79%)

Total	33,726	11,994 (35.56%)

### Animals and sampling procedures

Dogs were sampled in houses where human VL patients were diagnosed during the past 5 years, and in houses within 300 meters of patient houses. Local health workers accompanied the research team to the houses of VL patients and neighboring houses where consent was obtained for the sampling of dogs.

All sampled dogs were monitored for clinical signs compatible with canine leishmaniosis including exfoliative dermatitis, skin ulceration, onychogryphosis, lymphadenomegaly, splenomegaly and poor body condition with emaciation. Blood from all dogs was collected by cephalic or jugular venipuncture in EDTA and clot tubes for serum. Dogs with suspected clinical signs were sampled by fine needle aspirations of lymph nodes if lymphadenomegaly was present, and of the spleen when splenomegaly was palpated. Blood in EDTA was spotted on sterile filter paper for PCR, and blood in clot tubes was centrifuged and separated for serum to be used for serology. Lymph node and spleen aspirates were spotted on filter paper for PCR and in some cases also seeded in medium for parasite culture.

Samples were collected from dogs during three field trips in July of 2007, April 2008 and September 2008. The study in animals was approved by the Isaev Institute, a branch of the Uzbekistan Health Ministry, and the local health authorities in Namangan.

### ELISA serology, PCR, and parasite culture

Serum anti-leishmanial antibodies were determined by ELISA using crude leishmanial antigen as previously described [7]. Briefly, dog sera were diluted to 1:100 and incubated in *L. infantum *antigen-coated (MCAN/IL/1994/LRC-L639) plates for 1 hour. The plates were then incubated with Protein A conjugated to horseradish peroxidase and developed by adding the substrate 2,2-azino-di-3-ethylbenzthiazolihne sulfonate (ABTS) (Boerhinger Manneheim, Germany). Each plate was read at wavelength 405 nm after the positive control canine serum reached a value between 0.95 and 1.0. A sample was considered positive if the optical density was 2.6 times higher than the standard deviation of the control group.

DNA was extracted from blood on filter paper, lymph node and spleen aspirates, and from parasite cultures using the phenol/chloroform method, and internal transcribed spacer 1 region (ITS1) PCR was used to amplify leishmanial DNA from the samples essentially as previously described [8]. Identification of *Leishmania *parasites at species level was achieved by sequencing the ITS1 PCR product [5].

Fine needle aspirates from the lymph node and spleen of dogs were seeded into rabbit blood-agar NNN semi-solid as described by Abdeen et al. [9]. For mass culture, parasites were grown in Schneider's *Drosophila *Medium containing 10% fetal calf serum in 25 ml flasks. All cultures were maintained at 26°C.

### *Leishmania *strain typing

*Leishmania *strains cultured from dogs were typed by multilocus enzyme electrophoresis (MLEE) using a 15 enzymatic system and starch electrophoresis [10]. DNA samples from these strains were also analyzed for variation in 14 multilocus microsatellite sequences shown to be highly discriminatory within *L. infantum *[11,12]. This multilocus microsatellite typing (MLMT) used the primers sequences, PCR conditions and approaches for data evaluation previously described [5].

### Statistical analysis

Chi-square test was used for determining statistical significance. For all analyses statistical significance was indicated by a p-value < 0.05. Analysis was performed by the WinPepi™ statistical package.

## Results

A total of 162 dogs were tested for *Leishmania infection*. The distribution of dogs by village and results of diagnostic tests are shown in Table 2. Forty-two dogs (25.9%) had clinical signs suspected of canine leishmaniosis and fifty one (31.5%) were sero-positive. ITS-1 PCR (Table 2) was performed for 116 dogs using blood and 10 were positive (8.6%). PCR on lymph node and spleen tissues were carried out only on dogs with suspected clinical signs. Twenty five of 36 dogs (69.4%) with lymphadenomegaly were positive by lymph node PCR, and 15 of 25 (60%) dogs with splenomegaly were PCR-positive by spleen PCR. When combining results from blood and tissue PCR, 40 dogs (29.6%) were PCR-positive (Table 2). *Leishmania *were cultured from lymph node or spleen aspirates of 10 dogs.

**Table 2:** The numbers of dogs surveyed for leishmaniosis in each village, dogs with clinical signs suspected of the disease (clinical suspects), seropositive, PCR positive in blood or tissues, and positive by culture.

Village	No. of dogs sampled	Clinical suspects	Serology Positive	PCR positive of total number tested by PCR	Culture
Chodak	89	23	30 (33.7%)	24/77 (31.2%)	6
Oltinkan	26	7	5 (19.2%)	7/22 (31.8%)	2
Gulistan	40	8	13 (32.5%)	7/30 (23.3%)	1
Chorkesar	7	4	3 (42.9%)	2/6 (33.3%)	1

Total	162	42 (25.9%)	51 (31.48%)	40/135 (29. 6%)	10

Dogs from all four villages were positive by serology, PCR and culture. The overall rates of canine seropositivity in the different villages were similar and no association was found between seropositivity and village, dog age, or dog gender.

A significant association was found between the presence of suspected clinical signs of leishmaniosis and positive serology (p < 0.001). The positive predictive value, e.g. the likelihood that a dog with compatible clinical signs will be seropositive, was 60.5%. Twenty four dogs out of the 119 asymptomatic dogs (20.2%) were seropositive. Thus, the negative predictive value of clinical signs (i.e. percentage of asymptomatic dogs that were seronegative) was 79.8%.

**Figure 3:**
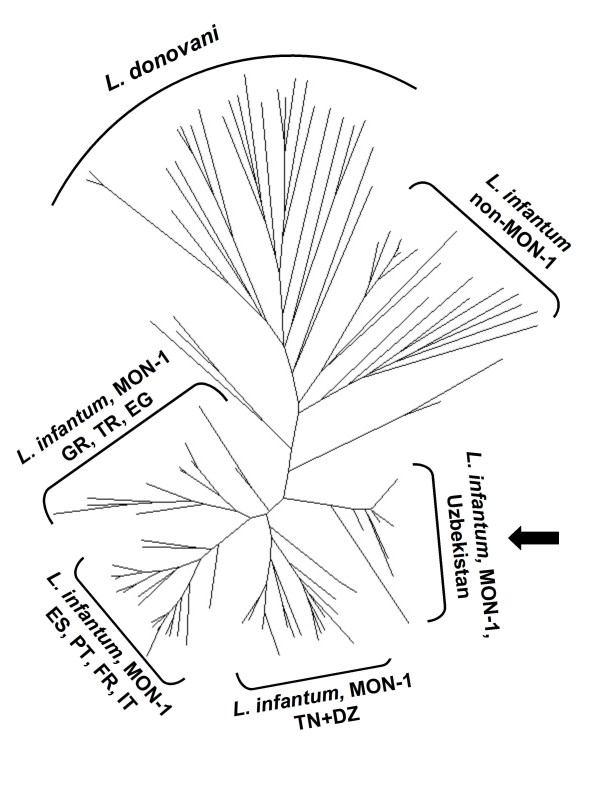
**Genetic analysis of *Leishmania infantum *isolates from the study area**. Neighbor-joining tree indicating that strains from Uzbekistan represent a unique genetic group of *L. infantum *MON-1. This unrooted tree was inferred from Dps (proportion of shared alleles) distance measure calculated for the data of 14 microsatellite markers. The microsatellite profiles of the Uzbeki strains (arrow) were compared to those of *L. infantum *MON-1 from Mediterranean foci of zoonotic VL, of *L. infantum *non-MON-1 strains and *L. donovani *strains of different origins (Alam et al. 2009). Strains with identical profiles appear only once in the tree. DZ = Algeria, TN = Tunisia, ES = Spain, PT = Portugal, FR = France, IT = Italy, GR = Greece, TR = Turkey, EG = Egypt.

Eight *Leishmania *strains isolated from dogs were typed by MLEE and MLMT. Both analyses revealed that the strains belong to the most common zymodeme of *L. infantum*, i.e., MON-1. By MLMT, all dog strains had the same genotype and were identical or highly similar to the strains isolated from humans in the study area. The microsatellite profiles of the canine and human strains of *L. infantum *from Uzbekistan characterized earlier [5] and in this study were compared to those of MON-1 strains from other geographical regions. The Uzbeki strains differed from other strains of *L. infantum *MON-1 and formed a distinct genetic group as shown in the Neighbour-joining tree based on the genetic distance (proportion of shared alleles measure) between the different profiles (Figure 3).

## Discussion

The Namangan region in Uzbekistan is an endemic region for human VL as indicated by the high numbers of patients and seropositive children reported from this area during 2006-2008 [4,5]. Although VL is a reportable disease in Uzbekistan which must be notified to the health authorities, the number of human cases is probably underestimated because of the lack of an efficient reporting system and appropriate diagnostic capabilities. This study has found that a high proportion of dogs are infected in the disease focus. Dogs harbor the same unique *L. infantum *MON-1 strain group identified in infected children from this region [5]. The fact that *L. infantum *was the causative agent of canine infection with typical clinical signs, and also of human infection affecting only infants clinically, suggests that a zoonotic form of VL similar in epidemiology to Mediterranean VL is present in Uzbekistan.

The unique *L. infantum *MON-1 strain group found in the Namangan region differs by MLMT from European, Middle Eastern and North African MON-1 strains. It has been, therefore, suggested that the Uzbeki strains have been circulating in Central Asia for a long time rather than having been introduced recently [5]. This supports the assumption that the Namangan focus is stable and that the disease is likely to have been present in this area for decades.

A significant association was found in this study between the presence of clinical signs compatible with canine leishmaniosis and seropositivity. However, a high proportion of dogs, 20.2%, were seropositive without clinical signs of infection, in agreement with previous surveys on canine leishmaniosis in endemic areas [13,14]. As found in other studies on canine leishmaniosis, this indicates that dogs in this region are infected asymptomatically and may serve as peri-domestic reservoirs for infection even without showing signs of infection [15,16]. The presence of clinical signs compatible with leishmaniosis can be helpful for the detection of some of the clinical cases, but it is not sufficient for diagnosis as some cases are infected without clinical signs, and others have non-specific clinical signs such as lymphadenomegaly, splenomegaly and epistaxis that can be caused also by other infectious diseases or inflammatory processes associated with other conditions [17].

The sensitivity of the PCR technique used in this study was limited due to the fact that it was done on blood in most dogs, which is considered less sensitive than lymph node, bone marrow or spleen [18,19]. The ITS-1 PCR used is also less sensitive than kDNA PCR, however it is useful for determining the *Leishmania *species causing the infection [20]. The ability to determine the species of the infecting agent is of particular importance in areas such as the Namangan region where *P. sergenti*, the major sandfly vector of *Leishmania tropica*, is present [3], as this species is able to cause VL in humans and dogs, in addition to being a major cause of human cutaneous leishmaniasis [21-23].

The seroprevalence rate of the dogs studied in the Namangan region is among the highest found in surveys of canine populations in endemic regions, with 8.1% found among dogs in the area of Madrid, Spain, 26% found in Malllorca, Spain, and 29.6% in the south of France [24,13,14]. In comparison to studies in other foci of VL, the Namangan region foci is located in an area where there is little awareness among the local population of the fact that this disease is zoonotic and associated with canine infection. No prevention of sand fly bites to dogs using topical insecticides in collars or spot on formulations is practiced as these measures are not available and cost-prohibitive. Vaccines against canine leishmaniosis could potentially also be effective in decreasing canine and human infection [25], as the Namangan focus is of limited geographic size and a vaccine campaign could reach the majority of households and domestic dogs living in the focus.

## Conclusions

In conclusion, widespread canine infection with a unique strain of *L. infantum*, also present in infected humans, has been found in the Namangan region of Uzbekistan. The pattern of disease appears to be zoonotic and similar in epidemiology to the Mediterranean form of VL. Preventative measures involving protection of dogs from becoming infected by sand fly bites or limiting the transmission of infection to sand flies from infected dogs could be helpful in decreasing the disease incidence in humans.

## Competing interests

The authors declare that they have no competing interests.

## Authors' contributions

DAK coordinated the studies in Uzbekistan, participated in sampling the dogs and in carrying out the molecular and serological assays. SAR was responsible for planning the study in Uzbekistan and participated in the field studies. ENP participated in the design of the study and in the field studies. AW participated in the design of the study and in the filed studies. RMN participated in the field studies and carrying out the diagnostic assays. VIP participated in the field studies and carrying out the diagnostic assays. AAF participated in the field studies and carrying out the diagnostic assays. AN participated in carrying out molecular assays. EK participated in the study design and the statistical analysis. MZA performed the MLMT analysis. LFS participated in the field studies, isolation and culture of *Leishmania *isolates. CLJ participated in the design of the study, field studies, and analysis of isolates. GS coordinated the project, participated in the design of the study and in the field studies, analyzed the genetic data and supervised the performance of the diagnostic assays. GB participated in the design of the study, coordinated the canine studies, participated the field studies, and drafted the manuscript. All authors have read and approved the final manuscript.
